# Effect of a high surface-to-volume ratio on fluorescence-based assays

**DOI:** 10.1007/s00216-012-5770-8

**Published:** 2012-02-12

**Authors:** Radoslaw Kwapiszewski, Karina Ziolkowska, Kamil Zukowski, Michal Chudy, Artur Dybko, Zbigniew Brzozka

**Affiliations:** Department of Microbioanalytics, Faculty of Chemistry, Warsaw University of Technology, Noakowskiego 3, 00-664 Warsaw, Poland

**Keywords:** Lab-on-a-chip, Microfluidics, Optical detection, Fluorescence, Fluorescein, Surface-to-volume ratio

## Abstract

In the work discussed in this paper, the effect of a high surface-to-volume ratio of a microfluidic detection cell on fluorescence quenching was studied. It was found that modification of the geometry of a microchannel can provide a wider linear range. This is a phenomenon which should be taken into consideration when microfluidic systems with fluorescence detection are developed. The dependence of the linear range for fluorescein on the surface-to-volume ratio was determined. Both fluorescence inner-filter effects and concentration self-quenching were taken into consideration. It was found that inner-filter effects have little effect on the extent of the linear range on the microscale.

**Figure:**
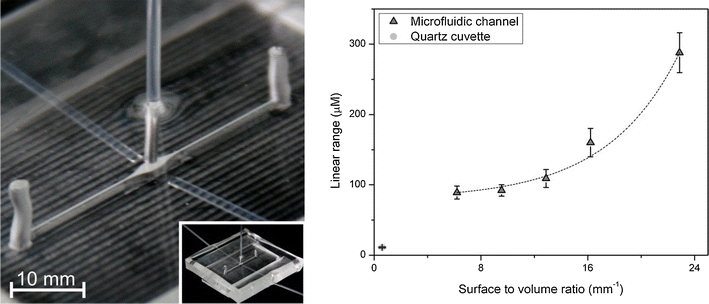
Dependence of the linear range on surface-to-volume ratio in microfluidic detection.

Dependence of the linear range on surface-to-volume ratio in microfluidic detection.

## Introduction

Today's widespread interest in microfluidics is mostly motivated by the possibility of exploitation of new phenomena present at the microscale. Some laws are not valid at the micrometer scale and, in consequence, fluid behavior can be totally different [1]. The downscaling leads to increased surface-to-volume ratio [2]. In the microdomain, surface tension and viscosity dominate over gravity and inertia. Electrokinetic pumping, surface tension-driven flows, electromagnetic forces, and acoustic streaming are effects that usually have no affect on macroscopic assays, whereas at the microscale they are fundamental [3]. The unique properties of the microdomain make it irreplaceable in many applications, e.g. for mimicking the in vivo environment [4] or for achieving high-resolution separations in capillary electrophoresis because of the flat velocity profile of electroosmotic flow [5].

Study of microfluidic systems for applied analytical purposes in chemistry, biochemistry, and life science has recently increased remarkably [6, 7].The challenge is the development of analytical methods compatible with the microscale. The smaller sample volumes reduce the number of analytes detected and hinder detection [8]. Low-volume detection within microfluidic chips is commonly performed by use of fluorescence, absorbance, and chemiluminescence [9]. Among these optical methods, fluorescence, which is highly sensitive and selective and relatively easy to integrate on microfluidic chips is still readily selected for measurements. Hence, gaining knowledge on fluorescence detection at the microscale and exploration of effects of new phenomena on fluorimetric measurements seem to be very important to avoid mistakes during development of new procedures and strategies. In the work discussed in this paper, the effect of a high surface-to-volume ratio (*S*/*V*) on fluorescence-based assays was investigated. It was found that increasing *S*/*V* by modification of microchannel geometry can significantly affect the linear range for fluorophores.

## Experimental

### Geometry and fabrication of μDCells

**Fig. 1:**
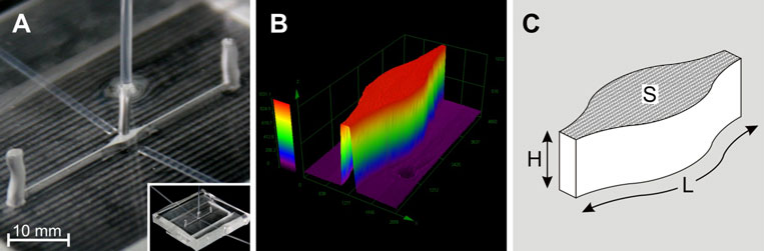
Construction of a μDCell: (**A**) photograph of the fabricated μDCell with optical fibers; (**B**) profile of a PMMA master of a 900 μm high μDCell; (**C**) schematic diagram of a μDCell with characteristic dimensions: *H* = 100, 150, 200, 300, 600 or 900 μm, *L* = 5 mm, and *S* = 3.48 mm^2^

Six microfluidic detection cells (μDCells) with different *S*/*V* ratios were designed, fabricated, and used in the experiments. Each μDCell was fabricated in poly(dimethylsiloxane) (PDMS) by the replica molding technique using a micromilled poly(methyl methacrylate) (PMMA) master (Fig. 1A). The μDCell was formed by widening a 150 μm wide microchannel up to 1200 μm (Fig. 1B). Dimensions of the μDCells (Fig. 1C) were measured using a laser confocal microscope (Lext; Olympus) to calculate the *S*/*V* ratios. To obtain structures with different *S*/*V* ratios the height of μDCells was changed. The *S*/*V* ratios 5.1, 6.2, 9.5, 12.9, 16.2, and 22.9 mm^−1^ were obtained for μDCells 900, 600, 300, 200, 150, and 100 μm high, respectively.

### Positioning of optical fibers

**Fig. 2:**
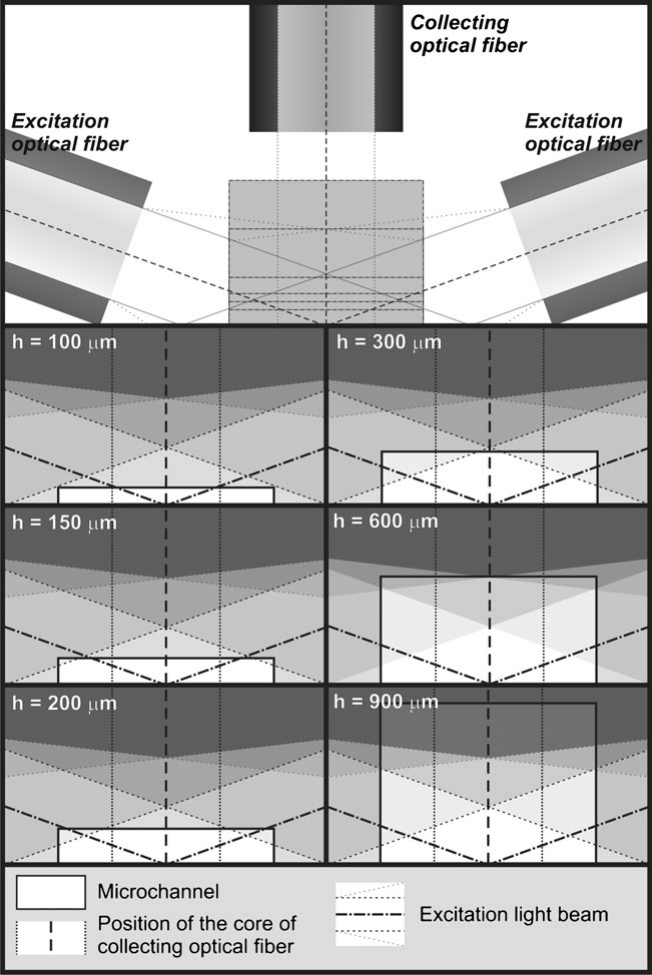
Schematic view of illumination of μDCells with different *S*/*V* ratios. It was observed that reducing the height (*h*) of μDCells resulted in shift of linear range toward higher concentrations of fluorescein

The μDCell was connected to a spectrofluorimeter (FluoroMax-3; Jobin Yvon) by use of quartz optical fibers (600/950 μm, NA = 0.22). The fibers were embedded in PDMS during molding. A specially designed holder for optical fibers enabled the same arrangement of optical fibers to be obtained in each μDCell. We found the optimum arrangement was two exciting fibers placed at a 20° angle to the surface and one collecting fiber placed orthogonally to the microchannel (Fig. 2). Each exciting fiber was 800 μm from the μDCell, whereas the collecting fiber was 1,200 μm from the bottom of the μDCell. In this arrangement the risk of transmitting excitation light to the detector is minimized. When the exciting fibers are placed at an angle >20° the collecting fiber cannot be placed so close to the μDCell, and, as a result, the detected fluorescence signal is low. When the fibers are placed at an angle <20° the fluorescence intensity of background increases, and analytes at low concentrations cannot be detected.

### Fluorescent compound

Investigations were conducted with sodium fluorescein diluted in 0.01 mol L^−1^ phosphate-buffered saline (PBS), pH 7.4. Fluorescein, rhodamine, coumarin, cyanine, pyrene, and their derivatives are the fluorophores most commonly used, e.g. in cell biology or flow cytometry.

## Results and discussion

Fluorophores with a small Stokes shift, for example fluorescein (*λ*_ex_ = 494 nm, *λ*_em_ = 521 nm) are particularly sensitive to concentration quenching [10]. High fluorescein concentrations result in self-quenching because of such interactions as radiative and non-radiative transfer and excimer formation [10]. Self-quenching is one phenomenon affecting the linear dynamic range, determination of which is a principle of fluorescence-based quantitative assays. Another phenomenon that may affect the linear range is inner-filter effects [11]. A primary inner-filter effect appears in concentrated solutions (absorbance >0.01) and means that fluorescence is not uniformly distributed in the cell [11]. The excitation radiation is mostly absorbed by the fluorophore or other chromophores as it enters the cell. Secondary inner-filter effects occur when the emitted fluorescence can also be absorbed by an appropriately absorbing component in the solution.

Investigation of the effect of μDCells’ *S*/*V* ratios on fluorescence quenching were started by measurement of the fluorescence intensity of fluorescein solutions using a typical spectrofluorimetry cuvette that was illuminated centrally and observed at a right-angle. The stability of the fluorescence signal of fluorescein over time was checked. Measurements were performed for concentrations of fluorescein ranging from 0.01 to 500 μmol L^−1^. Above a concentration of 20 μmol L^−1^ a decreasing fluorescence intensity signal was observed. The maximum linear range was up to 11 μmol L^−1^ (correlation coefficient *R* ^2^ > 0.97). Measurements of the fluorescence intensity of fluorescein were also performed using an off-center geometric arrangement^1^ of a 1 cm × 1 cm cuvette. Off-center illumination reduces the path length, and is generally used to reduce inner-filter effects [10]. In this case, a decrease of the fluorescence intensity signal was observed above the concentration 30 μmol L^−1^, and the maximum linear range was up to 14 μmol L^−1^ (data not shown). The results obtained confirmed the occurrence of inner-filter effects. However, the change of the linear range for both analyzed cases is slight, whereas for chemical compounds with a larger Stokes shift the change may be different by one order of magnitude [10].

**Fig. 3:**
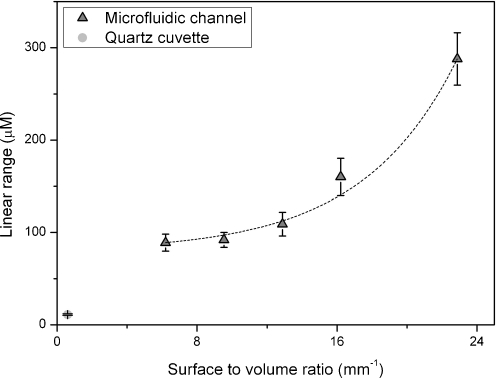
Dependence of the linear range on surface-to-volume ratio in microfluidic detection. The exponential growth model was assumed and fitted to the data points

The next step was to determine the calibration curves for fluorescein by using the fabricated μDCells with different *S*/*V* ratios. Each μDCell was tested using the same solutions of fluorescein within the range 0.01–500 μmol L^−1^. Each experiment was repeated five times. The linear ranges for the 900, 600, 300, 200, 150 and 100 μm high μDCells were up to 88, 89, 92, 109, 160, and 288 μmol L^−1^, respectively. The same linear ranges were obtained at different excitation intensities. These results deviate substantially from macroscopic results. The excitation intensities required for cuvette-based measurements are normally much less than the excitation intensities applied in optical fiber-based systems. Here, to eliminate additional effects that might occur because of the different excitation intensities in the cuvette and in the μDCell, a neutral gray filter was used for the cuvette-based measurements. It was, moreover, observed that reducing of the height of μDCells resulted in shift of linear range toward higher concentrations of fluorescein. The wider linear range was observed for μDCells with higher *S*/*V* ratios. This enabled determination of the dependence of the maximum range of linearity on the surface-to-volume ratio (Fig. 3). There are at least two possible explanations of this phenomenon: stronger inner-filter effects or probe-to-surface interactions effecting fluorescence quenching. A closer look at the fully illuminated 300 or 600 μm high μDCell and the partially illuminated (approx. 60%) 900 μm high μDCell (Fig. 2) was needed to estimate the effect of the inner-filter effects, which would have been strongest when comparing results between these μDCells. However, the results show there is no significant change of the linear range for μDCells 300, 600, and 900 μm high. On the other hand, significant extension of the linear range was observed when the height of a μDCell was reduced from 150 to 100 μm (i.e. increasing the *S*/*V* ratio 1.5-fold). Both μDCells were fully illuminated (Fig. 2). This observation led to the conclusion that the effect of changing the linear range might be attributed to probe-to-surface interactions. Performing similar experiments using surface-modified microfluidic detection cells could evaluate probe-to-surface interactions and confirm our hypothesis. The effect of increasing *S*/*V* ratio on the efficiency of chemical reactions has already been reported [12]. It was observed that microchannels with higher *S*/*V* ratios required higher polymerase concentrations to achieve PCR reactions of the same efficiency, but no further studies on this effect have been performed. In fluorescence detection, concentration self-quenching depends on collisions between molecules of a fluorophore. Increasing the *S*/*V* ratio increases the probability of molecule-to-cell wall collisions with no energy transfer. However, this hypothesis requires further investigation.

## Conclusion

The effect described should be taken into consideration when developing microfluidic systems utilizing fluorescence detection. The effect may cause significant divergences between results obtained on the micro and macro scales even if the same procedure is used. The effect is particularly important for assays based on fluorescence quenching, e.g. study of molecular interactions [13], determination of intracellular enzyme activity [14, 15], or even determination of the diffusion coefficient of oxygen in membranes [16]. Fluorescence intensities are proportional to concentration over a limited range of optical densities only. If the concentration of a sample is outside the linear range, sample dilution is necessary. However, dilution may cause changes in solvation, conformation, bonding, degree of association, and other chemical events [11]. Hence, to avoid sample dilution a number of approaches are used, e.g. use of different geometric arrangements for observation of fluorescence or use of mathematical corrections [10]. The results presented here show that use of miniaturized devices can also be an alternative means of obtaining a wider linear range, and for detection of compounds at high concentrations.

## Footnotes

1 The cuvette was shifted by 3 mm along the *X* and *Y* axes to reduce the path length
